# A comprehensive simulation framework for evaluating U.S. lung allocation policies

**DOI:** 10.1007/s10729-026-09797-z

**Published:** 2026-11-07

**Authors:** Hyunwoo Shin, Sait Tunc, Xi Chen, John M. Reynolds

**Affiliations:** 1 Grado Department of Industrial and Systems Engineering, Virginia Tech, 1145 Perry Street, Blacksburg, VA 24061 USA; 2 Department of Medicine, Division of Pulmonary, Allergy, and Critical Care Medicine, Duke University School of Medicine, 40 Medicine Circle, Durham, NC 27710 USA

**Keywords:** Lung transplantation, Organ allocation policy, Discrete-event simulation, Simulation modeling, Policy evaluation, Model calibration and validation

## Abstract

The rapidly evolving landscape of lung allocation policy, including the recent adoption of the Continuous Distribution framework, demands simulation tools that are both flexible and computationally efficient. Existing lung allocation simulators are often difficult to adapt to new policy designs and can be computationally burdensome when large numbers of replications are needed for uncertainty quantification and optimization. This paper introduces the Modular Lung Allocation Policy Simulation Model (MoLAPS), a high-fidelity, discrete-event simulation framework designed to address these limitations through a modular policy architecture and parallel execution. MoLAPS integrates detailed patient and donor data, dynamic medical status updates, and a predictive organ-offer acceptance model. It is implemented with parallel computing support, substantially reducing runtimes for multi-replication and longer-horizon experiments and enabling the exploration of large-scale policy design spaces. The model features a modular architecture that isolates policy components, including allocation, acceptance, and prioritization rules, allowing policymakers to easily evaluate existing and proposed allocation policies, or hypothetical alternatives. A surrogate-based calibration procedure aligns simulation outputs with national organ transplant data, improving empirical validity. MoLAPS is rigorously validated using prediction intervals, quantile-quantile plots, and Chi-squared tests. Simulation results closely align with real-world data across key performance indicators, including transplant rates, waitlist mortality, and organ nonuse. MoLAPS provides clinicians, policymakers, and researchers with a timely, open, and extensible platform for policy evaluation. By enabling realistic, data-driven assessments of lung allocation strategies, it supports more equitable, efficient, and evidence-based transplant policy decisions.

**Supplementary Information:**

The online version contains supplementary material available at https://doi.org/10.1007/s10729-026-09797-z.

## Highlights

 

- We introduce Modular Lung Allocation Policy Simulation Model (MoLAPS), a new simulation tool that mirrors every step of the U.S. lung allocation system and runs much faster than existing models.
- MoLAPS closely matches national data, offering a reliable baseline for testing new transplant policies.
- The model separates key decision rules into modules, making it easy to adapt as new policies, like Continuous Distribution, are introduced, or as new data becomes available.
- Policymakers, transplant programs, and clinicians can use MoLAPS to test how changes in allocation rules affect waitlist deaths, post-transplant survival, organ nonuse, average travel distance and regional equity measures.
- By improving realism and efficiency, MoLAPS helps transplant leaders make better, faster, and more informed decisions.

## Introduction

Lung transplantation is a vital, life-saving intervention for patients suffering from severe, end-stage lung diseases [1]. Given the critical nature of these conditions, timely access to donor lungs is essential for survival. However, the allocation of these scarce resources presents significant operational and ethical challenges. Despite advancements in organ procurement methods, surgical techniques, and postoperative care, the demand for lung transplants continues to exceed the available supply, resulting in prolonged waiting times and increased mortality among patients on the waiting list. For example, in 2024, 3,710 new candidates were added to the lung transplant waiting list, while only 3,340 deceased donor lungs were transplanted, leaving nearly 1,000 patients still awaiting transplantation [2]. This persistent imbalance underscores the pressing need for efficient, equitable, and effective organ allocation policies to optimize the utilization of available donor lungs.

Allocating deceased donor lungs is inherently complex, as it involves balancing multiple, often competing factors, including medical urgency, expected post-transplant survival, geographic disparities, and logistical constraints. To address these complexities, the Lung Allocation Score (LAS) system was introduced in 2005 to provide a more objective, data-driven basis for prioritizing transplant candidates. The LAS risk models were subsequently revised and implemented in 2015 (following OPTN Board approval) to improve risk adjustment and calibration, including for important subpopulations such as candidates with pulmonary arterial hypertension [3]. The LAS assigns each candidate a numerical score ranging from 0 to 100, calculated based on two primary survival probabilities: the waiting list survival probability over the next year (WLAUC) and the one-year post-transplant survival probability (PTAUC) [4]. The LAS thus prioritizes candidates according to both urgency and potential transplant benefit, incorporating additional factors such as geographic proximity, age, and blood type compatibility to ensure logistical feasibility and biological compatibility. Guided by four ethical principles—patient autonomy, beneficence, non-maleficence, and justice—the LAS framework aims to maximize patient outcomes while ensuring fair and equitable distribution of scarce resources [5].

Despite significant improvements introduced by the LAS, several limitations remain. Specifically, the LAS struggles to adequately capture dynamic changes in patient health status, leading to calibration errors that can result in inaccurate prioritization and suboptimal resource allocation [6]. Additionally, geographic disparities persist despite recent adjustments, and ischemic exposure remains insufficiently addressed by the LAS framework. Although both warm ischemia time (WIT) and cold ischemia time (CIT) affect graft viability, WIT is generally considered the more biologically injurious component of ischemic exposure, whereas prolonged CIT remains an important logistics-related risk [7, 8]. Consistent with the broader geographic sharing implemented in 2017, empirical analyses report increased travel and longer ischemic times; for example, mean ischemic time increased from 5.33 to 5.53 hours following the shift to the 250-NM circle policy [9], and the fraction of recipients with total ischemia time <4 hours declined from 24.2% (DSA-first era) to 17.6% (DSA-free era) alongside increases in donor–recipient travel distance and air transport [10]. In parallel, nonuse of recovered lungs remains nontrivial and has increased in the post-2019 period (e.g., 8.1% in 2021 and 9.0% in 2022, with 2023 at a similar level) [11]. While this increase may partly reflect broader donor utilization and procurement [12], the level of nonuse remains a meaningful concern in a supply-constrained system. Furthermore, prioritizing the sickest patients often leads to increased postoperative complications, prolonged hospitalizations, and elevated healthcare costs [13]. The national data indicate that adult lung waitlist mortality (deaths while listed per 100 patient–years) has declined over the past decade but remained approximately 13.3 deaths per 100 patient–years in 2023 [12]. These ongoing challenges underscore the necessity of continuous policy evaluation and refinement.

Recognizing these shortcomings, the U.S. organ allocation system has been gradually transitioning toward the Continuous Distribution framework since 2023. Continuous Distribution aims to eliminate rigid geographic boundaries and incorporate a more comprehensive set of criteria, including medical urgency, expected post-transplant outcomes, donor-recipient compatibility, and logistical considerations, into a unified Composite Allocation Score (CAS) [3]. By adopting a multi-factorial approach, CAS seeks to improve both equity and efficiency by prioritizing candidates with the greatest medical need and the highest expected benefit, while reducing geographic disparities in access [14]. While encouraging, this aggregate decline coexists with persistent heterogeneity and evolving practice, underscoring the need for policy evaluation under changing conditions. However, even with CAS, several issues persist, such as the highly nonlinear medical-urgency component potentially prioritizing candidates who may be too ill for transplantation, unintended disadvantages to specific patient groups (e.g., blood-type O candidates), and inadequate donor–recipient lung-size matching criteria [15–20]. These complexities highlight the critical need for a flexible, adaptable modeling framework to systematically evaluate, refine, and optimize allocation policies before their widespread implementation.

Optimizing lung allocation policies requires a holistic examination of the entire transplant system, as its components are highly interdependent. The complexity of balancing medical urgency, donor-recipient matching, and organ viability necessitates using sophisticated tools to evaluate various policy parameters in a controlled and dynamic environment. Simulation-based evaluation and optimization offers a robust approach to tackling these intricate challenges. The Scientific Registry of Transplant Recipients (SRTR) previously introduced the Thoracic Simulated Allocation Model (TSAM) [21], which has been instrumental in studying organ allocation policies. However, the model, last updated in 2015, is now outdated in terms of both policies and technological capabilities. Additionally, the model is distributed to the public as a black box, limiting its flexibility to implement any significant changes to the allocation system or to evaluate non-standard performance metrics such as logistics outcomes or geographic disparities, making it inadequate for addressing evolving needs in lung transplantation policy analysis. As lung allocation policy shifts from a classification-based system to the more nuanced score-based Continuous Distribution framework, the limitations of TSAM become more pronounced. Its current architecture is not optimized for the contemporary demands of policy evaluation, particularly in terms of computational efficiency [22]. This poses a significant barrier, as Continuous Distribution requires precise tuning of each policy component to ensure equitable and efficient organ allocation. As a result, there is a clear need for a more advanced, adaptable, and computationally efficient simulation model to support the evolving landscape of lung allocation policies and ensure accurate, timely decisions.

Addressing these critical needs, this study presents the Modular Lung Allocation Policy Simulation Model (MoLAPS). MoLAPS accommodates the complexities of modern lung allocation policies, featuring modular policy components and parallel computing to enable efficient, flexible evaluations of allocation strategies. These advancements facilitate contemporary optimization techniques by allowing independent adjustments of policy components, supporting development and refinement of advanced frameworks like Continuous Distribution. Recent policy discussions have also highlighted allocation out of sequence (AOOS), in which organs are offered or placed outside the policy-defined match sequence [23, 24]. Because AOOS departs from the standard match-run logic, it introduces an additional layer of operational complexity that may materially affect how allocation policy functions in practice. A flexible simulation framework such as MoLAPS can support future evaluation of these deviations by comparing policy-compliant match runs with alternative offer pathways and quantifying their effects on allocation outcomes. Furthermore, MoLAPS can integrate sophisticated theoretical mechanisms, such as incentive-based frameworks at candidate or transplant-center levels, for nuanced policy analyses. Leveraging parallel computing, MoLAPS significantly reduces computational burdens typical in large-scale optimization, setting a new standard for efficiency and scalability in organ allocation research.

### Policy evolution for lung allocation

The U.S. lung allocation system, managed by the Organ Procurement and Transplantation Network (OPTN), is responsible for ensuring equitable and efficient organ distribution using sophisticated donor-recipient matching algorithms and continuous outcome evaluation. To improve fairness and effectiveness, the U.S. Department of Health and Human Services issued the ‘Final Rule’ in 2000, mandating regular reviews and adaptation of the allocation system to medical advancements and changing patient needs [25].

A key component of this effort was the LAS system, which was in effect from 2005 until its replacement by the Continuous Distribution framework in 2023. It prioritizes lung transplant candidates based on medical urgency and post-transplant survival potential by assigning each patient a score between 0 and 100 calculated as:

1 $$\begin{aligned} LAS = \frac{100\times (PTAUC - 2\times WLAUC + 730)}{1095}, \end{aligned}$$

where WLAUC and PTAUC represent expected one-year survival on the waiting list and post-transplant, respectively, based on clinical factors, including age, functional status, body mass index, ventilation status, creatinine, bilirubin, and other relevant demographic and medical variables (see [4] for the details of the WLAUC and PTAUC formulation).

Prior to 2023, the U.S. lung allocation system determined patient priority using specific classifications that considered several donor- and patient-related factors such as age, geographical location, blood type compatibility, and medical urgency. Within each classification, candidates were ranked according to their LAS, with ties broken by total waiting time on the waiting list (see [4] for an archived version of the allocation policy). Geographical constraints play a critical role in organ accessibility, viability, and logistical costs [5]. Initially, the allocation system utilized Donor Service Areas (DSAs), regions assigned to organ procurement organizations. However, DSAs created geographic disparities due to variations in size, population density, and donor availability [9]. Two years after the major revisions to the LAS risk models, another major policy update was implemented in 2017 in response to a lawsuit in New York addressing geographic inequities [3]. This update eliminated DSAs and replaced them with fixed-distance concentric circles centered around the donor hospital, ranging from 250 nautical miles to nationwide coverage. It aimed to create a more equitable system by standardizing the geographic considerations in organ allocation, ensuring that candidates closer to the donor hospital received higher priority while allowing for broader organ sharing beyond local areas.

While the introduction of the LAS system and the 2017 update represented significant advancements in lung allocation [26], several limitations have become apparent. The LAS framework has been shown to suffer from calibration errors, particularly in underestimating survival probabilities for high-risk patients on the waitlist and overestimating post-transplant survival for those with the highest LAS scores. These inaccuracies result in suboptimal allocation of resources and adverse transplant outcomes for certain patient groups [6]. Furthermore, the prioritization of critically ill patients frequently leads to higher postoperative complications, prolonged hospitalizations, and increased healthcare costs, particularly among patients who require advanced interventions, such as extracorporeal membrane oxygenation (ECMO) [13]. Although the introduction of fixed-distance concentric circles reduced some geographic disparities, it did not fully address all aspects necessary to optimize allocation efficiency and equity. Specifically, this geographic allocation system did not adequately account for important factors such as varying population densities, logistical complexities associated with travel, and regional differences in donor availability [6, 27, 28].

To overcome these limitations, the CAS was introduced in 2023, marking the transition to the Continuous Distribution framework [29]. Unlike the rigid, classification-based LAS system, the CAS integrates multiple factors into a single continuous scoring system, considering medical urgency, expected post-transplant survival, donor-recipient compatibility, patient access, and geographic location. By holistically evaluating these dimensions, the CAS seeks to reduce waiting list mortality, improve long-term patient outcomes, and enhance access for challenging-to-match candidates. This integrated scoring system is recognized as more flexible and adaptable compared to previous frameworks, thereby enabling a more equitable and efficient allocation of donor lungs (see [29] for further details).

However, despite these notable advancements, the CAS still exhibits significant shortcomings. First, its medical urgency component features a highly nonlinear scoring scale: candidates with an expected waiting list survival of one year or more receive fewer than 10 percent of the maximum urgency points, whereas patients expected to survive only a few days receive the full 100 percent [15]. This design emphasizes urgency, but it can concentrate priority among candidates at the extreme tail of short-term waiting list risk, underscoring the importance of systematic outcome monitoring and periodic recalibration to mitigate unintended consequences as practice and donor utilization evolve. Consistent with the need for such monitoring, OPTN’s two-year Continuous Distribution monitoring report summarizes similar short-term post-transplant survival patterns in pre- versus (vs.) post-policy comparisons and does not indicate a clear deterioration signal in removals for “death or too sick” among the sickest candidates, while emphasizing that continued surveillance is warranted as additional follow-up accrues [30]. Second, analyses conducted by the International Society for Heart and Lung Transplantation (ISHLT) revealed that initial CAS weighting unintentionally disadvantaged blood-type O candidates [16]. In response, the OPTN subsequently adjusted the weighting to restore equity. Nevertheless, ISHLT recommended revisiting simulation frameworks such as TSAM to guide and validate future adjustments effectively.

Another critical determinant of transplant success is appropriate donor–recipient lung-size matching [17, 18]. Currently, CAS primarily relies on patient height to infer lung size compatibility, which can be insufficient and imprecise. Recent studies have proposed alternative approaches that could enhance size matching accuracy and patient outcomes [19, 20]. Incorporating these novel strategies and integrating updated patient data into advanced simulation models, such as MoLAPS, could further refine and rigorously evaluate CAS policies. Collectively, these challenges highlight the necessity of developing and utilizing an openly configurable and computationally efficient simulation model, like MoLAPS, to systematically evaluate and optimize proposed refinements to CAS prior to their national implementation.

### Objectives and contributions

This paper introduces the MoLAPS, a comprehensive simulation framework explicitly designed to address contemporary challenges in lung allocation. MoLAPS is developed to evaluate and optimize lung allocation policies by analyzing a critical three-year transitional period (2017–2019). This interval captures the impact of the fixed-distance circle policy implemented in 2017, but precedes the introduction of the CAS.

MoLAPS addresses limitations of existing simulation models in flexibility, computational efficiency, and predictive accuracy. Specifically, the model integrates detailed patient-level clinical and demographic information, employs dynamic survival analysis techniques for patient status updates, and utilizes sophisticated logistic regression methods to simulate realistic organ-offer acceptance decisions. Its modular architecture enables individual policy components to be independently updated or replaced, offering a highly adaptable framework well-suited for the evaluation of evolving allocation policies, such as Continuous Distribution. Furthermore, MoLAPS leverages parallel computing to achieve substantial gains in computational speed, thus enabling practical use of advanced optimization techniques, which are crucial for thoroughly assessing complex, multi-dimensional allocation scenarios.

The framework supports policymakers in refining allocation strategies, reducing rates of organ nonuse, and promoting a more equitable and efficient distribution of scarce donor lungs. Additionally, the transparent and configurable nature of MoLAPS facilitates rigorous evaluation and iterative refinement of policy parameters, thereby minimizing unintended consequences when implementing novel lung allocation mechanisms. To improve accessibility for researchers and practitioners, we provide a Dockerized runtime distribution together with GitHub-based documentation [31]. The container bundles the required dependencies and preprocessing scripts so users can reproduce results and run policy experiments without platform-specific configuration.

The remainder of the paper is structured as follows. In §2, we provide an in-depth review of existing literature on simulation modeling for organ allocation, highlighting prior methodologies, their limitations, and opportunities for improvement. §3 details the MoLAPS framework, including the integration of contemporary lung allocation policies, statistical methods for patient status updating, and decision-making processes. In §4, we describe our rigorous verification and validation processes, demonstrating the model’s accuracy, robustness, and reliability through detailed comparisons with empirical data and comprehensive calibration procedures. Finally, §5 summarizes the main findings, discusses their implications for optimizing lung allocation policies, and suggests promising avenues for future research aimed at further improving the efficiency, equity, and effectiveness of lung transplantation systems.

## Literature review

Simulation modeling is widely used in healthcare operations to analyze complex, stochastic systems and support decision-making [32]. In organ transplantation, discrete-event simulation (DES) is particularly well suited to represent the dynamic interactions among candidate arrivals, organ availability, clinical evolution, and sequential allocation decisions [33]. By enabling counterfactual evaluation of allocation strategies without disrupting real-world operations, DES has become a standard tool for assessing policy tradeoffs in scarce-resource settings, and can also support upstream procurement and distribution planning under uncertainty [34]. Early applications focused on specific organs and established the feasibility of full-system simulation for transplantation policy analysis. For example, [35] developed an early simulation model of kidney transplantation dynamics, and the UNOS Liver Allocation Model (ULAM) enabled national-scale evaluation of liver allocation policies under alternative scenarios [36].

Subsequently, simulation modeling evolved to address allocation efficiency and equity at the national scale. Notably, the UNOS Kidney Allocation Model (UKAM) provided a robust framework to analyze national kidney allocation policies, facilitating systematic assessment of policy alternatives based on empirical data [37]. [38] further employed Monte Carlo simulations to evaluate evidence-based strategies in kidney transplantation, explicitly comparing traditional point-based systems against efficiency-driven and distributive fairness algorithms. Extending these methods to liver transplantation, [39] developed a clinically detailed DES model incorporating patient-specific data, enabling nuanced assessments of policy changes on outcomes such as transplant survival and waiting times. Complementary liver studies similarly used DES to evaluate waiting list management and national allocation rules, quantifying equity-efficiency trade-offs across alternative policies [40, 41].

Simulation models have also been used to evaluate allocation policies through broader welfare and resource-allocation lenses. For example, patient-level kidney simulators have quantified trade-offs in quality-adjusted life years and costs under alternative deceased-donor allocation rules [42], and related work has examined how clinical decision-making may shift under rapidly changing conditions, such as transplant-timing strategies during the COVID-19 pandemic [43]. Because allocation policies are ultimately realized through center decisions to accept or decline offers, many simulators incorporate explicit accept–decline modules; even relatively parsimonious, interpretable logistic models can capture key determinants of acceptance and materially influence predicted transplant throughput and organ nonuse [44]. In parallel, simulation has supported economic evaluations of policy alternatives. For instance, [45] introduced LiverSim to evaluate the cost-effectiveness of liver allocation policies in the United Kingdom. Their analyses indicated that prioritizing patients who were less severely ill, rather than those who waited the longest, could enhance overall cost-effectiveness. Similarly, [40] demonstrated that allocation policies favoring younger or less critically ill patients could improve both cost-efficiency and outcomes, underscoring the simulation’s critical role in balancing equity and efficiency through rigorous quantitative evaluation.

Recent simulation studies have increasingly focused on refining patient-level clinical variables to better represent the intricate dynamics of transplantation. Incorporating detailed medical factors, such as functional status, comorbidities, and clinical scoring systems, has enabled more tailored policy analyses. For example, [46] incorporated detailed comorbidity profiles into a liver transplant simulator to better reflect clinical decision-making, and [47] used a Markov-based framework that integrates dialysis duration and MELD scores to evaluate simultaneous liver–kidney transplantation policies. Simulation models have also been used to study ethical and geographic disparities; [48] employed agent-based modeling to examine multiple listing and found evidence of local inequities and potential disadvantages for smaller centers. More recently, calibrated microsimulation for older kidney candidates has linked SRTR-based risk equations to long-term outcomes across policy scenarios, illustrating potential survival gains even with lower-quality organs and providing a template for subgroup-specific analyses [49].

At the system-design level, geography-aware optimization and evaluation tools complement patient-level simulators. For example, recent work studies geography-aware policy design through heterogeneous sharing radii [50] and through location-allocation formulations that examine capacity siting and sharing rules, and quantify their implications for access and outcomes [51]. Rule-testing platforms such as KARS compare jurisdiction-specific kidney allocation rules on real or synthetic waiting lists [52], while other simulation studies evaluate modifications to compatibility or matching criteria within existing allocation algorithms [53]. Regional end-to-end DES has also integrated donation, logistics, and allocation to evaluate access, waiting-list deaths, and the impact of siting additional transplant centers [54], and complementary work has examined transportation and coordination challenges in transplantation networks [55]. Advances in computational methods have further enabled simulators that are both flexible and efficient for large-scale policy evaluation; [56] developed a faster DES platform for kidney transplantation, and [57] introduced COMET, an open-source, modular framework that relies on synthetic populations to support plug-and-play experimentation across organs, including a lung module.

MoLAPS complements existing transplant simulators with a different design emphasis. It is built for high-fidelity, U.S. lung-specific policy evaluation using real OPTN listing, waitlist-update, and donor data, while explicitly implementing OPTN match-run logic for the 2017–2019 regime. The simulator is implemented in C/C++ with MPI-based parallelization and is calibrated to reproduce multiple system-level benchmarks, enabling repeated large-scale runs needed for sensitivity analysis and simulation-optimization. More broadly, the framework is designed to address practical gaps that often limit the use of policy simulators for contemporary lung-allocation questions: limited transparency for methodological extensions, difficulty updating policy components as rules evolve, and computational burden that restricts extensive scenario exploration. To address these challenges, we develop a modular architecture that isolates policy and behavioral components (e.g., allocation logic, acceptance behavior, and dynamic status evolution) so that they can be updated independently, and we couple this with a scalable parallel implementation.

## Model

Effective simulation modeling of the U.S. lung transplant system is essential for analyzing the outcomes of policy adjustments and for identifying optimal allocation strategies for deceased donor lungs. A well-structured model must simultaneously balance multiple, often competing objectives, including reducing mortality on the waiting list, shortening waiting times, and maximizing post-transplant survival outcomes. Our primary goal with MoLAPS is to enhance computational efficiency, flexibility, and integrability, without sacrificing accuracy or detail. MoLAPS employs a modular architecture that distinctly separates critical components of the simulation–including the lung allocation policy module, patient-status update models, and organ offer acceptance predictions. This modularity facilitates independent updates, replacements, or improvements within individual modules, ensuring adaptability to policy evolutions or methodological advancements.

We selected C/C++ for MoLAPS implementation due to its superior computational speed, flexibility, and extensive library support. To optimize computational performance, we integrated the Armadillo C++ library, well-known for its efficient matrix algebra capabilities [58]. Moreover, MoLAPS utilizes the OpenMPI library to harness parallel computing across multiple CPUs, substantially accelerating computations and enabling efficient simulation across many replications [59]. In our parallel computing implementation, a designated master core distributes simulation tasks to multiple worker cores, significantly enhancing overall performance during multi-replication scenarios.

The modular design of MoLAPS is specifically engineered for seamless integration of advanced statistical models, including logistic regression models for organ offer acceptance and dynamic survival analysis for patient-status updates. This design ensures adaptability, permitting individual modules to be efficiently revised in response to new clinical insights, data availability, or methodological advancements. Such flexibility significantly broadens the scope and applicability of MoLAPS, enabling it to robustly support evolving lung allocation frameworks and advanced theoretical analyses.

The remainder of this section is organized as follows: In §3.1, we provide an overview of the simulation model. §3.2 and 3.3 present detailed descriptions of the statistical models incorporated within the simulation model. In §3.4, we outline the procedure for calibrating MoLAPS. Finally, §3.5 reports a runtime comparison between MoLAPS and TSAM.

**Fig. 1:**
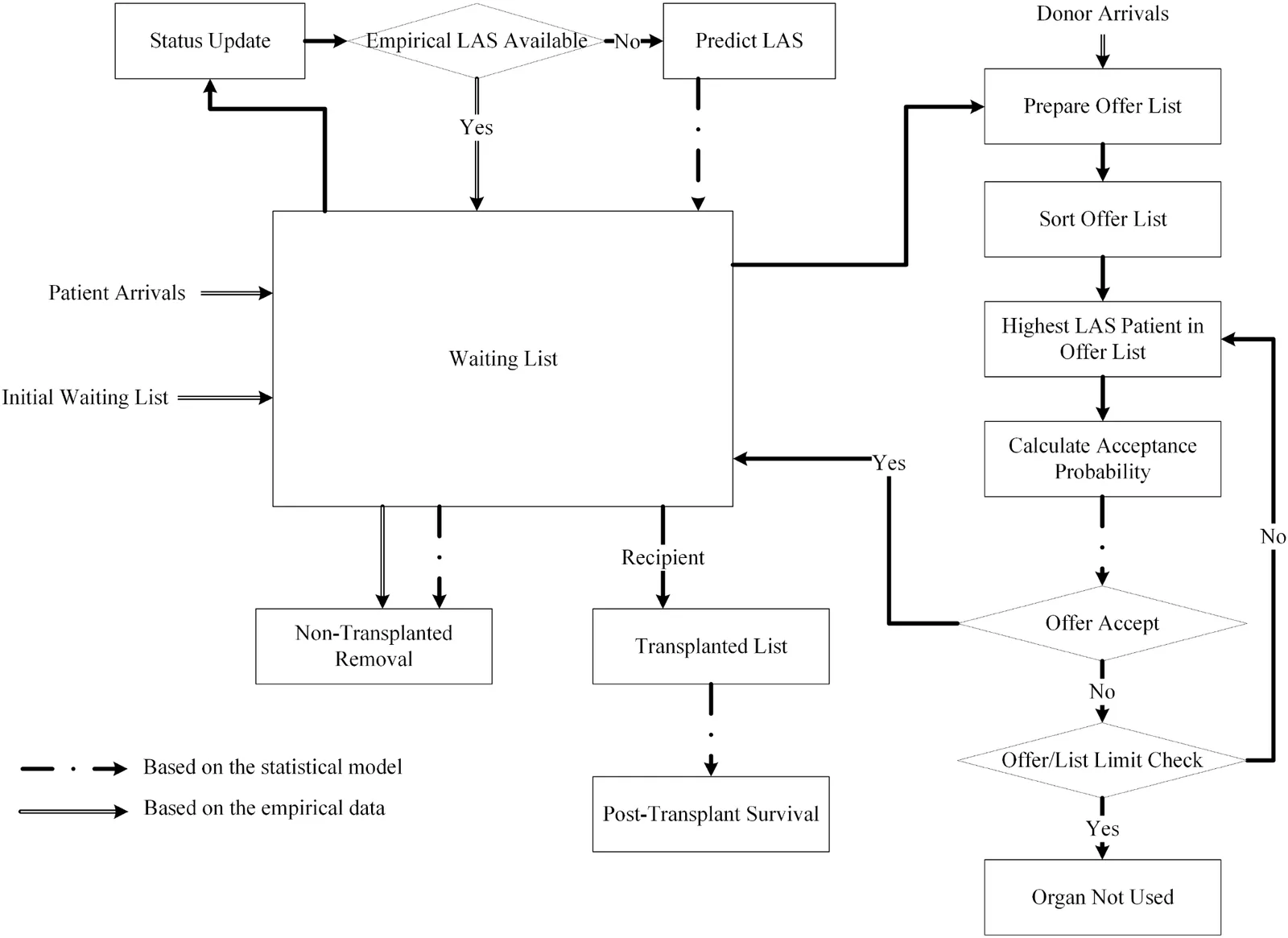
An overview of the simulation model

### Overview of the simulation model

Figure 1 illustrates the flowchart of MoLAPS, which is based on a discrete event simulation framework. The model comprises five main components: patient arrivals, organ arrivals, patient status updates, offer decisions and post-transplant updates, and patient removals. The details of each component are described below.

#### Key events in the simulation

Five event types drive the model: patient arrivals, patient status updates, donor arrivals, patient removals, and post-transplant updates. These events are carefully designed to replicate the dynamics of the real-world lung allocation process. Each event is summarized below; the statistical sub-models that govern updates, offers, and outcomes are detailed in §3.2–3.3.

- *Patient Arrivals:* The patient arrival events involve adding new patients to the waiting list along with their detailed initial clinical and demographic information. New candidates are introduced exactly as they appeared in OPTN data, preserving realistic arrival rates and clinical heterogeneity, without relying on assumed theoretical distributions. Because MoLAPS directly replays the historical listing records, temporal patterns in listing activity, including diagnosis-specific trends, seasonal variations, or year-to-year changes, are inherited from the data. The model therefore introduces no additional assumptions about arrivals; stochastic variation arises from downstream components such as organ-offer acceptance, patient-status evolution, and non-transplant removals.
- *Patient Status Updates*: The status update event refreshes patient information throughout the simulation, reflecting changes over time, such as age, LAS, and lab values. These updates represent patients’ evolving status on the waiting list. While empirical data provides a foundation for these updates, MoLAPS introduces elements of randomness to capture inherent uncertainties in patient status updates. For patients who received a lung transplant in the real system but remain in the simulation past their actual transplant date, statistical models are used to update their LAS and predict their eventual removal reasons, such as death, medical unsuitability, or improvement in condition, if they do not receive a transplant in the simulation. Detailed information on these updates is provided in §3.2.
- *Donor Arrivals and Organ Offers*: Donor arrival is a pivotal event that triggers the organ allocation (match run) process, leading to subsequent offer decisions and potential transplants. Donor arrivals follow the historical records, but the actual allocation can vary depending on assignment rules and acceptance models within the simulation. Upon donor arrival, the model first updates the waiting list and then constructs the offer list. This list is filtered based on the eligibility of candidates for the lungs from the given donor and classified based on OPTN policies [4] (see §3.3 for details), considering various factors such as geographical location, blood type match, and candidate age. The filtered offer list is then sorted by priority class according to allocation rules. Each donor can provide a maximum of two lungs, so the allocation process is repeated for each donated lung. If an organ is not placed after exhausting the offer list within the “offer limit" constraints, the lung is recorded as unused. Further details on the organ offer decision-making process are provided in §3.3. The modular design allows the offer module to be swapped for a future CAS-based rule without altering other components.
- *Patients’ Removal*: Patients may leave the system in the simulation due to two main reasons: transplantation or non-transplant-related removals. Reasons for non-transplant removals include death, significant detriment (medical unsuitability for transplantation), or improvement in condition. Given the uncertainty inherent in the simulation, discrepancies may arise where patients recorded as having received a transplant in the empirical data might need to be removed for non-transplant reasons in the simulation after a certain period. This handling of mismatches between simulated and empirical cases is further detailed in §3.2.
- *Post-Transplant Outcomes*: Survival estimates are modeled using the SRTR post-transplant patient survival model, implemented as a Cox proportional hazards framework with donor and recipient covariates and their interactions [60]. Post-transplant outcomes include graft failure, subsequent relisting for re-transplantation, and patient death. Recipients who die post-transplant permanently exit the system. Re-transplantation, which accounts for approximately 3% of lung transplants nationally [61, 62], is represented by relisting recipients who experience graft failure; once relisted, these candidates re-enter the waiting list and are allocated organs under the same simulation logic as primary transplants.

#### Input data

MoLAPS uses real-world datasets to model and simulate patient and donor arrivals, drawing on historical data from the OPTN over three years, from January 1, 2017, to December 31, 2019. This timeframe is chosen to exclude the COVID-19 era while capturing the recent major changes in U.S. lung allocation policy, ensuring the data reflects a relatively typical and up-to-date operational environment. The raw dataset is processed, cleansed, and formatted using R scripts for use in the simulation model.

MoLAPS focuses on adult patients, aged 18 and above, who are listed solely for lung transplantation. This focus is due to the use of the standard lung allocation rules for these patients, as opposed to those requiring multiple organ transplants (e.g., lung and heart), who are treated under different protocols. The dataset includes 10,279 transplant candidate records and 8,289 deceased donor records. Each patient’s location is specified by a center code with corresponding latitude and longitude, while donor locations are also geo-coded. The distance between donors and transplant centers is calculated using the Haversine formula, which approximates geographic distance based on latitude and longitude.

MoLAPS utilizes three primary types of input data for accurately simulating the lung allocation process:

(i) *Waitlisted Candidate Data:* This data includes patient demographics, such as age, gender, weight, height, ethnicity, and medical information, such as diagnosis group, initial listing date, end date, and reasons for removal from the waiting list for lung transplant candidates (see Table 1 for a summary of key candidate characteristics). For patients with missing basic information (e.g., height or weight), values are interpolated using averages from the same diagnosis group, gender, and similar weight or height.
(ii) *Historical Waitlist Updates Data:* This dataset captures dynamic updates in patient data during their time on the waiting list, including changes to the LAS, WLAUC and PTAUC (see Fig. S1 for the evolution of these scores over time for a set of representative patients). On average, there are 5.2 observations per patient, with a standard deviation of 5.86. The maximum number of observations for a patient is 66 (see Fig. S2 for a histogram of the number of updates). Within the simulation, the patient information is dynamically adjusted to reflect current status, ensuring the simulation accurately mirrors real-world scenarios.
(iii) *Deceased Donor Data:* This data includes detailed information about the demographics and medical characteristics of deceased donors from whom at least one lung was recovered for transplantation (see Table 2 for a summary of key donor characteristics).

The outputs of the simulation are organized into four comprehensive datasets: transplant recipients, non-transplant removals, yearly snapshots of the waiting list, and unused organs. These datasets are used to evaluate the overall performance, effectiveness, and implications of the simulation. We utilize R and Python scripts to analyze these outputs.

**Table 1:** Summary of transplant candidate input data

	Percent / Mean (Std)		Percent / Mean (Std)
N	10279	N	10279
Diagnosis Group		Previous Malignancy	
A	26.73%	No	92.42%
B	6.35%	Yes	7.58%
C	8.47%	Lung Surgery	
D	58.44%	No	96.88%
Gender		Yes	3.12%
Male	55.15%	History of Cigarette Use	
Female	44.85%	Yes	56.93%
Blood Type		No	43.07%
O	47.40%	Steroid Use	
A	37.35%	No	65.70%
B	11.41%	Yes	34.30%
AB	3.84%	Prior Cardiac Surgery	
Ethnicity		No	95.91%
White	76.00%	Yes	4.09%
Black	10.60%	Initial Age	56.89 (12.86)
Hispanic	9.70%	Initial Height	168.76 (10.16)
Asian	2.83%	Initial Weight	73.99 (16.67)
Native American	0.42%	Initial BMI	25.81 (4.55)
Multiracial	0.35%	mPAP	27.46 (11.21)
Pacific Islander	0.10%	O2 Requirement	5.70 (6.06)
ECMO in Last 72 Hours		Cardiac Output	5.31 (1.46)
No	93.96%	Central Venous Pressure	8.45 (4.04)
Yes	6.04%	FEV1% Predicted	41.53 (21.01)
Life Support		FVC% Predicted	50.15 (18.03)
No	93.58%	PCWP	10.61 (5.49)
Yes	6.42%	Six-Minute Walk Distance	722.55 (424.68)

Acronyms: Mean Pulmonary Artery Pressure (mPAP), Pulmonary Capillary Wedge Pressure (PCWP).

Units: mPAP (mmHg), O2 Requirement (L/min), Cardiac Output (L/min), Central Venous Pressure (mmHg), PCWP (mmHg)

**Table 2:** Summary of deceased donor input data

	Percent / Mean (Std)		Percent / Mean (Std)
N	8289	N	8289
Donor Blood Type		Donor Gender	
O	50.31%	Male	60.20%
A	35.96%	Female	39.80%
B	11.45%	Number of Lungs	
AB	2.28%	1	90.19%
Donor Ethnicity		2	9.81%
White	62.44%	History of Hypertension	
Black	17.06%	No	74.69%
Hispanic	15.66%	Yes	25.31%
Asian	3.25%	Risk for HIV	
Multiracial	0.84%	No	72.53%
Native American	0.51%	Yes	27.47%
Pacific Islander	0.24%	History of Cigarette Use	
DCD		No	91.84%
No	92.32%	Yes	8.16%
Yes	7.68%	History of Diabetes	
Chest X-Ray		No	90.77%
Yes	69.88%	Yes	9.23%
No	30.12%	HCV NAT Result	
Cause of Death (Donor)		Negative	97.07%
Head Trauma	37.46%	Positive	2.93%
Anoxia	33.51%	Age	35.80 (13.81)
Stroke	26.69%	Height	171.42 (10.37)
Other	1.85%	Weight	78.73 (18.87)
CNS Tumor	0.49%	PO2	385.11 (135.04)
Total Bilirubin	0.99 (1.39)	Creatinine	1.53 (1.69)

Acronyms: Donation after Circulatory Death (DCD), Hepatitis C Virus (HCV), Human Immunodeficiency Virus (HIV), Nucleic Acid Test (NAT).

Units: PO2 (mmHg), Creatinine (mg/dL), Total Bilirubin (mg/dL)

**Table 3:** Dynamically updated variables

Variable	Initial Value	Update Method
Age	Initial age	Calculated over time
PTAUC, WLAUC	Empirical data	Empirical data (if available), else prediction model
LAS	Empirical data	Empirical data (if available), else calculated
Bilirubin	Empirical data	Empirical data
Creatinine	Empirical data	Empirical data
Predicted FVC	Empirical data	Empirical data
Serum Creatinine	Empirical data	Empirical data

Predicted FVC: Predicted FVC Pre Bronchodilator

### Dynamic updates for patient status

In MoLAPS, patients undergo dynamic status updates on various characteristics and lab values while they remain on the waiting list. These updates include changes in age, LAS, and other relevant variables (see Table 3 for a detailed list of dynamically updated variables and their data sources).

The patient status is updated based on historical records if available. However, due to the inherent uncertainty in the simulation environment, situations not present in real-world data can occur, such as a patient remaining on the waiting list beyond their actual transplant date. In such cases, predicting dynamic changes in the LAS score is essential to represent accurately patient priority on the waiting list when empirical data is lacking. To address this, we employ a Markov chain modeling framework to predict the evolution of the WLAUC and PTAUC (see §A.2 for details), enabling dynamic updates to each patient’s LAS according to Eq. 1 throughout their time on the waitlist.

Another critical update is required for patients who received a transplant in reality but remain on the waiting list in the simulation past their actual transplant date. For these patients, we must estimate their expected survival duration on the waitlist and determine the likely reason for their removal if they do not receive a transplant within the simulation. To accomplish this, we first categorize patients into three groups based on their actual outcomes: (1) transplanted patients with censored waitlist survival times who require estimation of their remaining waitlist survival time and potential removal reason; (2) non-transplanted patients with observed waitlist survival times and known removal reasons; and (3) patients who remain waiting for transplantation at the end of the simulation horizon. For patients in groups 1 and 2, we employ a Cox regression model to estimate their 6-month, 1-, 3-, and 5-year waitlist survival probabilities (see §A.1 for model details). We then identify a non-transplanted patient whose survival probability vector is closest to that of the patient in question, as measured by Euclidean distance, ensuring that this matched patient has a longer observed waitlist survival and belongs to the same age category as defined by the OPTN [62]. The matched patient’s waitlist survival time and removal reason are then assigned to the simulated patient. This approach, inspired by predictive mean matching used to minimize bias in imputation methods [63, 64], ensures that patient statuses in the simulation closely reflect realistic clinical trajectories, thereby enhancing the accuracy, validity, and reliability of MoLAPS’s results.

Because waitlist removal reasons are center-reported and documentation practices may vary across transplant programs, these fields can be noisy at a granular code level. MoLAPS therefore uses removal reasons conservatively by aggregating non-transplant removals into a small set of clinically interpretable categories: *death*, *medically unsuitable*, and *condition improved*, with all remaining codes grouped as *other removal*. This aggregation limits sensitivity to idiosyncratic coding differences while preserving the key distinctions needed to characterize waitlist outcomes and support calibration and validation. These aggregated categories are used only for reporting and for matching system-level outcome counts, and we avoid interpreting fine-grained removal codes as ground truth at the individual level.

### Organ offer decisions

In the U.S. lung allocation system, organs are matched with potential recipients through a sequential matching procedure, commonly known as a match run. During a match run, organ offers are sent sequentially to a ranked list of eligible potential recipients until a match is found and the organ is placed, or until the organ’s viable window expires, resulting in nonuse. Eligibility for transplantation considers multiple factors, including blood type compatibility, patient-specific constraints (e.g., maximum acceptable donor age), and other donor-recipient interactions. Once eligibility criteria are satisfied, candidates are prioritized according to OPTN policies. Eligible patients are classified based on age, blood type compatibility, and the distance between the donor and transplant hospitals. Within each classification group, the candidates are further sorted by four hierarchical criteria: LAS (highest to lowest), total waiting time (longest to shortest), date and time of LAS variable update (earliest first) and LAS exception date (earliest first).

As the match run progresses, provisional offers are sent according to this prioritized list. Candidates who provisionally accept the offer are then required to provide a final acceptance or rejection within a predetermined timeframe. An essential factor during this sequential offering process is the CIT, i.e., the duration from clamping of the organ until its transplantation. As the number of offers increases, the cumulative CIT typically rises, potentially affecting organ quality and viability. Excessive CIT may render the organ unsuitable for transplantation, causing it to remain unused despite available recipients.

In MoLAPS, the filtered list of eligible patients, referred to as the offer list, is dynamically generated whenever a donor organ becomes available. Organ offer decisions are modeled using the SRTR’s medically detailed offer acceptance prediction model [60]. Specifically, the likelihood that a candidate will accept a specific organ offer is calculated using a logistic regression framework that incorporates detailed donor-patient interactions, patient and donor characteristics, and the patient’s relative rank within the offer list [60]. To simulate actual responses, we apply the Monte Carlo method, generating a uniform random number to determine acceptance or rejection. Formally, let $O_{ij}$ denote the binary outcome of offer acceptance (1 if accepted, 0 otherwise) for patient *i* and donor *j*, modeled as:

$$\begin{aligned} O_{ij}\sim \text {Bernoulli}(p_{ij}), \quad p_{ij}=logit^{-1}(\gamma ^{\intercal } X_{ij}), \end{aligned}$$

where $X_{ij}$ represents the vector of patient and donor characteristics and their interactions, and γ is the corresponding vector of regression coefficients. This process continues iteratively until an acceptance occurs–resulting in transplantation and initiation of post-transplant outcome modeling (see §3.1)–or the predefined offer limit is reached without acceptance, leading to organ nonuse.

**Table 4:** Calibration parameters for the offer response framework

Parts	Calibration Parameters
Offer acceptance	Intercept
prediction model	Coeff. for log(number of offers)
	Coeff. for log(number of offers), if offers ≥400
	Coeff. for log(waiting time +1), if waiting time ≥100
	Coeff. for log(waiting time +1), if waiting time ≥1000
Offer limit	Positive integer value

**Table 5:** Comparison of execution time between TSAM and MoLAPS (hh:mm:ss)

Simulation period	Number of replications	TSAM	Our Model	Speedup
1 year	1 replication	00:00:29	00:01:46	0.27×
	10 replications	00:05:28	00:01:57	2.80×
	20 replications	00:08:58	00:02:08	4.20×
	100 replications	00:44:51*	00:02:51	15.74×*
2 years	1 replication	00:01:05	00:03:30	0.31×
	10 replications	00:11:20	00:03:33	3.19×
	20 replications	00:18:17	00:03:58	4.61×
	100 replications	01:15:40*	00:05:03	14.98×*
3 years	1 replication	-	00:03:59	-
	10 replications	-	00:04:36	-
	20 replications	-	00:05:02	-
	100 replications	-	00:06:09	-

The times provided with a “*" are extrapolated using linear regression

Although the SRTR acceptance model provides robust predictions, some discrepancies between simulated results and observed organ offer acceptance patterns can occur. To address this, we included several parameters from the offer acceptance model within our calibration process, thereby improving alignment with empirical data. These calibration parameters are summarized in Table 4, with further details provided in §3.4.

Another critical component of the organ offer decision-making process is the *offer limit*, which approximates the viable window for a donor lung during the match run. Practically, CIT, organ retrieval timings, donor medical status, and response times to offers collectively influence this limit. To maintain computational simplicity, consistent with previous organ allocation simulation models [21, 65], we assume a constant offer limit. This constant limit is included as a calibration parameter, allowing adjustment to reflect observed outcomes more accurately. However, this simplifying assumption might introduce biases in estimating organ nonuse. Future work should address this limitation by allowing the offer limit to dynamically vary according to critical factors such as donor condition and accumulated CIT, thus enhancing the model’s realism and predictive capability.

### Calibration

Calibration is critical in ensuring that MoLAPS accurately reflects real-world lung allocation dynamics. Due to inherent assumptions in simulation models, such as simplified offer limits and approximations in statistical models predicting patient and donor behaviors, discrepancies inevitably arise between simulated and empirical data. The calibration process addresses these differences, aligning the model’s outputs with observed outcomes, thereby enhancing the model’s reliability for evaluating lung allocation policies. Our calibration approach adheres to principles outlined in [66] and employs iterative surrogate-based techniques detailed by [67].

Several challenges within our organ allocation simulation necessitate calibration. First, the simplified assumption of a fixed offer limit, defined as the maximum number of offers an organ can undergo before becoming unusable, can introduce potential biases. While computationally convenient, this assumption fails to capture real-world variability arising from factors like CIT and donor-specific conditions. Additionally, predictive statistical models for offer acceptance and patient outcomes rely on historical data and may not fully encapsulate all nuances inherent to clinical decision-making, further necessitating calibration.

The primary goal of our calibration process is to minimize discrepancies between the simulation outputs and real-world data. We formulate calibration as an optimization problem, adjusting six carefully selected input parameters (summarized previously in Table 4) based on their substantial influence on simulation outcomes. Calibration specifically targets four key metrics: (i) the average *number of offers before acceptance*, capturing allocation efficiency; (ii) the average *waiting time until transplantation*, indicating system timeliness; (iii) the average *number of unused organs*, highlighting inefficiencies in organ utilization; and (iv) the average *number of patients remaining on the waiting list* at the simulation horizon’s end, reflecting overall system demand. Further details of the calibration formulation and the iterative surrogate-based optimization procedure are deferred to Appendix A.4; the full algorithm is summarized in Algorithm S1.

### Computational performance and scalability

Table 5 compares the execution time of MoLAPS with TSAM across simulation horizons of one to three years and varying numbers of replications. Because TSAM supports at most 20 replications and a two-year horizon, its runtimes for 100 replications are estimated by linear extrapolation from the available runs (marked with an asterisk). For single-replication experiments, MoLAPS can be slower due to fixed overhead associated with parallel execution. However, as the number of replications increases, MoLAPS amortizes this overhead and achieves substantially lower total runtimes. In the settings most relevant for policy evaluation and simulation-based optimization, MoLAPS yields large speedups, reaching approximately a 15–16× improvement for 100-replication experiments in Table 5. These gains enable large replication budgets, longer horizons, and broad scenario sweeps that would otherwise be computationally prohibitive.

**Table 6:** Validation results for the simulation model: point-to-distribution case

Category	Original data	Simulation results		Relative error
	Count	Mean (HCI95)	95% PI	
TX	7352	7345.10 (4.57)	(7309.00, 7390.58)	-0.09%
Nonuse	622	620.90 (4.57)	(575.43, 657.00)	-0.18%
Non-TX	1607	1621.37 (4.15)	(1580.90, 1661.53)	+0.89%
Death	516	506.69 (2.66)	(475.43, 529.53)	-1.80%
Other removal	538	556.05 (2.58)	(527.48, 580.53)	+3.36%
Medically unsuitable	441	451.95 (2.68)	(423.00, 474.58)	+2.48%
Condition improved	112	106.68 (1.13)	(95.00, 116.52)	-4.75%
Last snapshot in the waiting list	1320	1332.54 (4.32)	(1290.28, 1371.53)	+0.95%

HCI95: half-width of 95% confidence interval, PI: prediction interval, TX: transplanted

## Model validation and performance evaluation

Ensuring the reliability and accuracy of MoLAPS is essential, particularly given the high stakes associated with organ allocation policies. Therefore, we conducted a rigorous verification and validation process. Verification ensures correct model implementation according to design specifications, while validation evaluates the accuracy of the model’s representation of real-world dynamics. Model validation is widely recognized as a critical component of healthcare decision modeling, where transparency, internal correctness, and comparison with empirical patterns are emphasized as key principles. Although discrete-event simulation studies often distinguish verification from validation, the healthcare decision-modeling literature commonly treats verification as a component of model validation [68]. For clarity, we describe verification activities first and then present our external validation analyses. Despite the recognized importance of these procedures, universally accepted standards for validating healthcare simulation models, especially for organ allocation systems, remain absent [69, 70]. Accordingly, our validation approach follows these general principles while adopting techniques tailored to the stochastic and event-driven nature of the lung allocation process.

### Challenges in simulation model validation

Traditional statistical tests, such as t-tests or Mann-Whitney tests, are often unsuitable for stochastic organ allocation simulators because assumptions such as normality or independence may not hold [71]. Specifically, each observed dataset is effectively a single realization of complex, random interactions, not necessarily representing the central tendency of all possible outcomes. Organ allocation simulations are inherently stochastic, producing a distribution of outcomes rather than a single deterministic result. Given the stochastic nature of MoLAPS, we employed validation techniques capable of capturing both central tendencies and variability inherent in the system.

Our validation strategy centers around two primary methods: (i) comparing individual real-world data points against the simulation-generated distribution of outcomes, and (ii) comparing the entire empirical distribution to that obtained through repeated simulation replications.

### Validation Framework

We structured the validation around several complementary analyses: verification of implementation, point-to-distribution and distribution-to-distribution validations.

#### Verification

As the first step in validation, we reviewed the MoLAPS implementation to detect and correct logical errors. Internal consistency checks were performed by carefully tracing patient-level trajectories throughout the simulation, ensuring accurate implementation of dynamic updates in patient status (e.g., LAS scores and eligibility). Additionally, we rigorously tested critical modules, such as the donor–recipient matching and organ offer decision processes, in isolation to confirm their correct behavior under controlled conditions.

#### Point-to-distribution validation

Point-to-distribution validation determines whether observed empirical data fall within the variability captured by multiple simulation runs. Specifically, we examined key system metrics including the number of transplanted patients, unused organs, patients removed from the waitlist without transplantation, and patients remaining on the waiting list at simulation conclusion. For each variable, we computed the relative error between the empirical value and the mean simulation output, along with a 95% prediction interval defined by the simulation distribution.

Table 6 summarizes these validation results. The selected variables encompass essential service metrics (transplantations), reneging outcomes (non-transplant removals), and queue statistics (remaining patients). Results indicate small relative errors for most metrics, demonstrating close alignment between simulated and real-world outcomes. Although some specific removal categories exhibit slightly higher relative errors due to small observed counts, the empirical values consistently fall within the simulation’s prediction intervals, validating the model’s robustness.

**Table 7:** Validation results for the simulation model: distribution-to-distribution case

Category	Original data	Simulation results		Relative error
	Mean (Boot 95% CI)	Mean (HCI95)	95% PI	
TX	133.95 (128.01, 140.04)	131.00 (0.45)	(127.26, 135.13)	-2.20%
Non-TX	433.14 (398.72, 469.26)	443.14 (1.58)	(427.49, 459.41)	+2.31%
Death	241.82 (206.02, 281.07)	225.65 (2.08)	(205.11, 244.82)	-6.69%
Other removal	568.00 (512.99, 625.95)	617.67 (2.94)	(589.68, 649.91)	+8.75%
Medically unsuitable	294.12 (244.78, 347.98)	299.95 (2.65)	(270.53, 320.65)	+1.98%
Condition improved	1214.13 (948.35, 1515.91)	1174.97 (11.74)	(1061.88, 1274.27)	-3.23%

HCI95: half-width of confidence interval with 95%. Boot 95% CI: bootstrap confidence interval based on 10,000 resamples. PI: prediction interval. Units: days

**Fig. 2:**
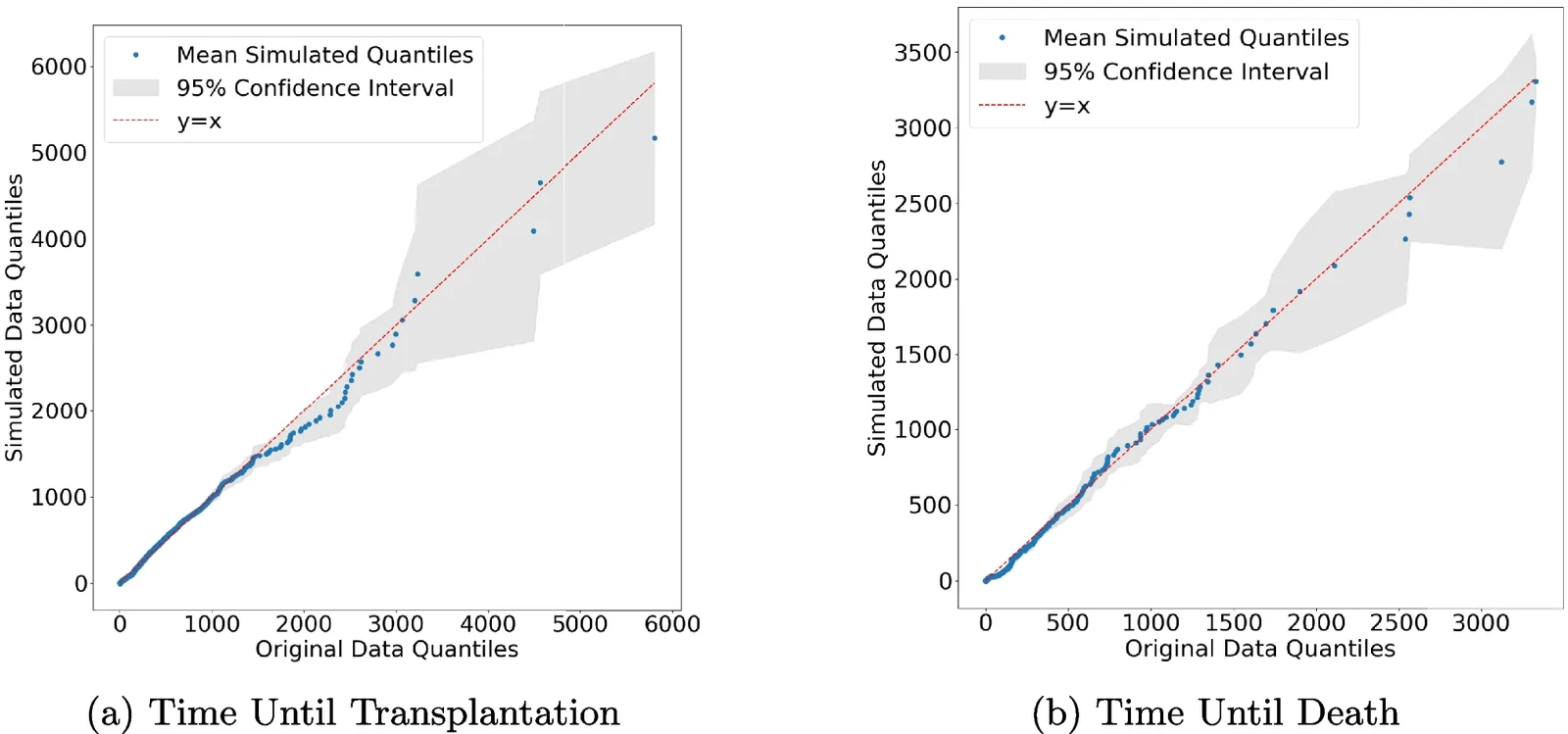
QQ plots for the waiting time of patients during the simulation horizon

#### Distribution-to-distribution validation

Distribution-to-distribution validation assesses whether the empirical waiting-time distribution is consistent with the distribution generated by repeated simulation replications. This comparison is particularly important for continuous time-to-event outcomes (e.g., time to transplantation, death, or other removals), where model fidelity depends on reproducing not only average waiting times but also the dispersion and tail behavior that drive clinically meaningful differences in access.

Table 7 summarizes results across six categories. Alongside the simulation mean and its 95% confidence interval across replications (HCI95), and the 95% prediction interval (PI) of the simulated outcomes, we also report 95% bootstrap confidence intervals (Boot 95% CI) for the empirical mean waiting times. The bootstrap intervals quantify sampling variability in the observed cohort, whereas HCI95 and PI reflect replication-level uncertainty and stochastic variability induced by MoLAPS. Across most categories, the empirical mean waiting time lies within the 95% prediction interval. The only exception is *other removal*, where the empirical mean falls slightly below the simulation PI, corresponding to a modest relative error of +8.75%. We interpret this deviation cautiously because *other removal* aggregates heterogeneous removal codes and can be sensitive to category composition and reporting variation. Moreover, the bootstrap 95% confidence interval for the empirical mean overlaps the simulated mean, indicating consistency once empirical uncertainty is accounted for.

Quantile–quantile (QQ) plots (Fig. 2) provide a complementary distribution-level check by comparing empirical quantiles with simulated quantiles averaged across replications; the shaded bands denote 95% confidence regions for the simulated quantiles. The empirical curves largely track within these bands, supporting MoLAPS’s ability to reproduce both the central tendency and the distributional characteristics of observed waiting-time outcomes.

**Table 8:** Multivariate validation: point-to-distribution case

Category	Original data	Bonferroni-adjusted PI
TX	7352	(7293.95, 7411.12)
Nonuse	622	(554.88, 672.05)
Non-TX	1607	(1556.26, 1672.91)
Death	516	(469.24, 533.76)
Other removal	538	(524.93, 583.69)
Medically unsuitable	441	(420.31, 484.52)
Condition improved	112	(91.62, 118.69)
Last snapshot in waiting list	1320	(1277.17, 1377.83)

PI: prediction interval. Bonferroni-adjusted PIs are marginal intervals constructed as described in the Multivariate Validation text, using $K{=}8$ jointly validated variables

**Table 9:** Multivariate validation: distribution-to-distribution case

Category	Original data	Bonferroni-adjusted PI
TX	133.95	(126.27, 136.30)
Non-TX	433.14	(425.16, 463.00)
Death	241.82	(201.27, 249.35)
Other removal	568.00	(583.41, 653.92)
Medically unsuitable	294.12	(264.96, 322.72)
Condition improved	1214.13	(1040.43, 1305.47)

PI: prediction interval. Bonferroni-adjusted PIs are marginal intervals constructed as described in the Multivariate Validation text, using $K{=}6$ jointly validated variables

#### Multivariate validation

Because simulation outputs are correlated, assessing them jointly requires controlling the overall (family-wise) coverage. To obtain a conservative joint check, we apply a Bonferroni adjustment to the marginal prediction intervals. For a set of *K* outputs and a target joint coverage of 1-α, we construct each marginal prediction interval at level 1-α/K. By the union bound, this construction provides joint coverage of at least 1-α. We use this criterion to assess joint reproduction of key system outcomes.

Tables 8 and 9 report the Bonferroni-adjusted prediction intervals for the point-to-distribution and distribution-to-distribution validation sets, respectively. In the point-to-distribution case (Table 8), all empirical values lie within the joint-adjusted intervals. In the distribution-to-distribution case (Table 9), all categories are covered except *other removal*, whose empirical mean falls slightly below the Bonferroni-adjusted lower bound. This single deviation is consistent with our univariate distribution-to-distribution results: *other removal* aggregates heterogeneous removal reasons and is therefore the most sensitive to category composition and reporting variation. Overall, these results provide evidence that MoLAPS reproduces the joint behavior of key outcomes, while also highlighting *other removal* as the primary category where residual mismatch remains.

**Table 10:** Validation results for the simulation model by year

Year	Category	Original data	Simulation results		Relative error
		Count	Mean (HCI95)	95% PI	
2017	TX	2342	2335.31 (2.42)	(2308.38, 2358.05)	-0.29%
	Nonuse	183	190.69 (2.42)	(167.95, 217.62)	+4.20%
	Non-TX	540	526.87 (2.08)	(508.48, 548.05)	-2.43%
	Death	182	172.10 (1.32)	(160.95, 185.05)	-5.44%
	Other removal	182	179.56 (1.29)	(167.95, 192.52)	-1.34%
	Medically unsuitable	139	135.39 (1.28)	(123.48, 148.00)	-2.60%
	Condition improved	37	39.82 (0.61)	(34.00, 45.00)	+7.62%
2018	TX	2422	2431.78 (2.71)	(2407.38, 2456.58)	+0.40%
	Nonuse	216	206.22 (2.71)	(181.43, 230.62)	-4.53%
	Non-TX	554	568.14 (2.97)	(535.00, 597.05)	+2.55%
	Death	199	191.91 (1.81)	(173.95, 209.05)	-3.56%
	Other removal	167	185.11 (1.70)	(166.43, 201.58)	+10.84%
	Medically unsuitable	147	150.98 (1.46)	(136.48, 163.00)	+2.71%
	Condition improved	41	40.14 (0.71)	(34.48, 48.00)	-2.10%
2019	TX	2580	2578.01 (2.45)	(2554.00, 2599.05)	-0.08%
	Nonuse	223	223.99 (2.45)	(202.95, 248.00)	+0.44%
	Non-TX	512	526.36 (2.68)	(502.00, 550.00)	+2.80%
	Death	135	142.68 (1.45)	(130.48, 156.52)	+5.69%
	Other removal	189	191.38 (1.70)	(173.48, 206.52)	+1.26%
	Medically unsuitable	154	165.58 (1.71)	(148.48, 180.52)	+7.52%
	Condition improved	34	26.72 (0.69)	(20.48, 33.00)	-21.41%

HCI95: half-width of confidence interval with 95%, PI: prediction interval

#### Yearly breakdown of results

To assess whether MoLAPS captures year-to-year fluctuations in donor supply, candidate mix, and acceptance behavior, we also validated key outcomes separately for 2017–2019 (Table 10). Across all three years, transplant volume is closely reproduced, with relative errors below 0.5%. For nonuse and overall non-transplant removals, the observed counts lie within the 95% prediction intervals in each year, indicating that remaining deviations are consistent with stochastic variability. For waitlist deaths, the model slightly underestimates 2017 and 2018 and slightly overestimates 2019, but in all three years the observed death counts remain within the corresponding 95% prediction intervals. At the subcategory level, *other removal* in 2018 and *condition improved* in 2019 show the largest relative errors; the observed counts lie within or close to the corresponding 95% prediction intervals. Overall, the yearly breakdown supports that MoLAPS reproduces both aggregate outcomes and temporal patterns with acceptable accuracy under stochastic uncertainty.

#### Goodness of fit for categorical variables

To assess the model’s accuracy in representing categorical characteristics that significantly influence allocation decisions (such as patient age group, ethnicity, diagnosis, and blood type), we employed Pearson’s Chi-squared tests for goodness-of-fit [72]. Results summarized in Tables 11 and 12 demonstrate consistency between simulation-generated distributions and real-world categorical distributions, with all p-values exceeding the significance threshold (0.05). These results underscore the simulation’s strength in accurately representing demographic and clinical diversity within the lung transplantation system.

**Table 11:** Number of transplanted patients by key categories

Grouped by	Type	Count		Percent (%)		P-value
		Actual	Simulation	Actual	Simulation	
Age	18–34	656	718.56	8.92	9.78	0.95
	35–49	859	978.59	11.68	13.32	
	50–64	3305	3369.54	44.95	45.87	
	65–74	2439	2204.35	33.17	30.01	
	75+	93	74.06	1.26	1.01	
Ethnic	Black	702	805.24	9.55	10.96	0.94
	Hispanic	647	703.16	8.80	9.57	
	Others	229	251.74	3.11	3.43	
	White	5774	5584.96	78.54	76.04	
Diagnosis group	A	1859	1889.76	25.29	25.73	0.98
	B	350	396.74	4.76	5.40	
	C	672	715.48	9.14	9.74	
	D	4471	4343.12	60.81	59.13	
Blood type	A	2892	2947.64	39.34	40.13	1.00
	AB	305	291.02	4.15	3.96	
	B	847	867.40	11.52	11.81	
	O	3308	3239.04	44.99	44.10

**Table 12:** Number of death in the waiting list by key categories

Grouped by	Type	Count		Percent (%)		P-value
		Actual	Simulation	Actual	Simulation	
Age	18–34	40	39.69	7.75	7.83	0.84
	35–49	60	40.15	11.63	7.92	
	50–64	249	260.11	48.26	51.34	
	65–74	162	161.04	31.40	31.78	
	75+	5	5.70	0.97	1.12	
Ethnic	Black	75	62.24	14.53	12.28	0.90
	Hispanic	63	58.82	12.21	11.61	
	Others	26	23.31	5.04	4.60	
	White	352	362.32	68.22	71.51	
Diagnosis group	A	92	74.95	17.83	14.79	0.88
	B	37	35.68	7.17	7.04	
	C	28	26.76	5.43	5.28	
	D	359	369.30	69.57	72.88	
Blood type	A	162	153.10	31.40	30.22	0.98
	AB	26	26.36	5.04	5.20	
	B	60	55.15	11.63	10.88	
	O	268	272.08	51.94	53.70

**Table 13:** Patient outcome proportions for temporal robustness

Category	2017–2019 Orig (%)	2017–2019 Sim (%)	2016 Orig (%)	2016 Sim (%)
Macro-level outcomes				
TX	71.52 (7352.00)	71.32 (7345.10)	54.00 (2222.00)	52.19 (2148.87)
Non-TX	15.63 (1607.00)	15.74 (1621.37)	13.20 (543.00)	18.65 (767.81)
LSWL	12.84 (1320.00)	12.94 (1332.54)	32.81 (1350.00)	29.16 (1200.79)
Micro-level outcomes (within Non-TX)				
Death	32.11 (516.00)	31.25 (506.69)	30.02 (163.00)	35.98 (276.26)
Other removal	33.48 (538.00)	34.30 (556.05)	36.28 (197.00)	31.49 (241.80)
Medically unsuitable	27.44 (441.00)	27.87 (451.95)	29.28 (159.00)	27.05 (207.72)
Condition improved	6.97 (112.00)	6.58 (106.68)	4.42 (24.00)	5.47 (42.03)

Percentages are shown with counts in parentheses. LSWL: last snapshot in the waiting list

#### External Validation and Cross-period Robustness

External validation is an important component of establishing model credibility [68]. For MoLAPS, however, strict out-of-sample validation under an *identical* allocation environment is not feasible. The simulator is calibrated to the 2017–2019 U.S. lung allocation environment, using nationwide OPTN data to represent the full system. Temporally, adjacent periods reflect materially different operating conditions: years prior to late 2017 follow the DSA-based geographic policy, whereas 2020 onward is affected by COVID-19 era disruptions and then subsequent redesign of the allocation policy in March 2023. However, comparisons outside this period are still informative about the performance of the historical configuration under changed operating conditions. We therefore report a *cross-period robustness* analysis using the 2016 cohort, the most recent complete calendar year entirely under the earlier DSA-based geographic policy, as an illustrative hold-out under a different policy environment. We supplement this analysis with a comparison using patient and donor records and waitlist updates from March 2022 through February 2023, the final 12 complete calendar months before the transition to CAS in March 2023. We selected this period to provide a more recent comparison while avoiding 2020–2021, when lung listing and transplantation activity were directly affected by COVID-19-related disruptions, and to remain entirely within the LAS-based allocation era. For this comparison, we retain the allocation configuration and calibrated parameters used for 2017–2019 (§A.5). Together, these analyses identify where agreement with outcomes outside the calibration period is maintained and where the historical configuration requires reassessment; they do not isolate the effects of individual policy or practice changes.

We examine the 2016 cohort to assess whether MoLAPS behaves sensibly outside its calibration window and captures the broad structural shifts associated with the earlier allocation environment, rather than to claim out-of-sample predictive accuracy under unchanged conditions. Table 13 compares (i) macro-level patient outcome proportions (TX / Non-TX / LSWL) and (ii) micro-level removal-reason composition within Non-TX. Table 14 summarizes Pearson Chi-squared goodness-of-fit tests for these distributions.

At the macro level, MoLAPS closely reproduces the 2017–2019 outcome distribution (Orig vs. Sim: $p{=}0.95$), consistent with the calibration target. As expected, the 2016 empirical macro distribution differs markedly from 2017–2019 (Orig 2017–2019 vs. Orig 2016: $p{<}0.01$), reflecting genuine system differences under the earlier DSA-based environment, including substantially higher LSWL and lower transplant proportions. When driven by 2016 inputs, MoLAPS moves in the same direction (2016 Orig vs. 2016 Sim: $p{<}0.01$), reproducing the large qualitative shift toward higher LSWL and lower transplant rates, while some level differences remain (e.g., Non-TX and death within Non-TX). We interpret these residual gaps as expected when applying a simulator configured for the post-2017 match-run structure to a pre-2017 policy environment, and we therefore treat this analysis as a robustness check rather than a definitive external validation.

**Table 14:** Summary of Chi-squared goodness-of-fit tests

Level	Comparison	$\chi ^2$	P-value
Macro	2017–2019 Orig vs. Sim	0.11	0.95
	2017–2019 Orig vs. 2016 Orig	778.88	<0.01
	2017–2019 Sim vs. 2016 Sim	619.07	<0.01
	2017–2019 Orig vs. 2016 Sim	630.00	<0.01
	2016 Orig vs. 2016 Sim	48.51	<0.01
Micro	2017–2019 Orig vs. Sim	0.58	0.90
	2017–2019 Orig vs. 2016 Orig	6.16	0.10
	2017–2019 Sim vs. 2016 Sim	5.93	0.11
	2017–2019 Orig vs. 2016 Sim	4.78	0.19
	2016 Orig vs. 2016 Sim	6.82	0.08

Macro-level tests use categories (TX / Non-TX / LSWL). Micro-level tests use removal reasons within Non-TX (Death / Other removal / Medically unsuitable / Condition improved)

At the micro level, the removal-reason composition within Non-TX is well preserved in the calibration period (Orig vs. Sim 2017–2019: $p{=}0.90$). In addition, the 2016 empirical and simulated micro-level compositions are not statistically distinguishable at the 5% level (2016 Orig vs. 2016 Sim: $p{=}0.08$), and the simulated 2016 composition is also not distinguishable from the 2017–2019 empirical composition (2017–2019 Orig vs. 2016 Sim: $p{=}0.19$). Taken together, these results suggest that, while macro-level outcomes appropriately shift across eras and cannot be expected to match exactly under a different policy environment, the core patient-pathway mechanisms generating non-transplant removals remain comparatively stable.

## Conclusion and future research directions

This study presents a novel simulation framework, MoLAPS, designed specifically for the evaluation and optimization of lung allocation policies in the United States. Recognizing significant limitations of existing models, particularly TSAM, MoLAPS substantially improves computational efficiency, adaptability, and integrability. By leveraging parallel computing and a modular architecture, our model not only achieves faster runtimes but also effectively accommodates the complexities associated with contemporary lung allocation frameworks, including the recent transition to the Continuous Distribution system.

A central contribution of our work lies in creating a comprehensive, modular simulation framework capable of realistically capturing key dynamics of lung allocation. The model seamlessly integrates patient and organ arrival data, dynamically updates patient statuses, and simulates detailed organ-offer decision processes within a discrete event simulation environment. Its parallel computing capability significantly reduces computational time compared to previous simulation approaches, a critical improvement facilitating extensive simulation-based optimization tasks. Furthermore, the modular design allows individual components–such as allocation rules, acceptance criteria, and patient-status updating algorithms–to be independently updated or replaced. This flexibility is especially valuable for accommodating evolving policy elements under the Continuous Distribution framework, enabling robust analyses of complex, multi-objective policy scenarios.

Rigorous validation underscores MoLAPS’s reliability. Using complementary checks, including relative errors, quantile-quantile comparisons, Chi-squared tests, and prediction-interval based assessments, we find close agreement between simulated outcomes and empirical data across key system metrics. As expected, agreement is less tight in smaller or sparsely represented categories, where both the empirical estimates and the simulated outcomes exhibit higher variability. In addition, the broad “other removal” category shows occasional larger year-specific deviations, which is likely driven by its heterogeneous composition, aggregating multiple non-transplant removals into a single outcome, and its sensitivity to center-level coding. Nevertheless, in these cases, prediction-interval and distributional checks indicate that most empirical values are covered, while a small number show modest deviations. At the aggregate level, the model closely reproduces national volumes and outcome composition, supporting overall model fidelity for policy evaluation and decision support.

Nevertheless, several limitations of MoLAPS should be acknowledged. First, the simulation currently assumes a constant offer limit to simplify complex factors affecting organ viability, such as CIT, WIT, and donor-specific conditions. While essential for computational tractability, this simplification may introduce biases, particularly in estimating organ nonuse rates. Second, the model focuses exclusively on adult patients listed solely for lung transplantation, excluding pediatric candidates and patients requiring multiple organ transplants. Expanding MoLAPS to incorporate these additional patient populations would enrich comprehensiveness but would also heighten complexity, given the specific rules and exceptions in the allocation of organs to such patients. Third, the simulation employs historical data to predict patient status changes and organ offer acceptances; however, these historical patterns may not fully capture future shifts in clinical practices or patient decision-making behaviors. As medical practices and allocation policies continue to evolve, periodic updates to statistical models and input data will be essential to preserve the accuracy and applicability of our model.

Furthermore, our calibration and validation focus on the stable pre-COVID period (2017–2019). System conditions changed markedly after 2019 due to pandemic-era disruptions that affected procurement logistics, critical care capacity, and donor evaluation practices, and were accompanied by temporal shifts in outcomes such as nonuse and waiting-list mortality. In addition, subsequent policy and practice changes, including the rollout of Continuous Distribution and evolving donor utilization (e.g., increased DCD and preservation technologies), may further alter acceptance behavior and outcome patterns relative to the 2017–2019 baseline. These considerations underscore that results in this paper should be interpreted within the pre-COVID calibration regime; as more post-policy data accrue and stabilize, MoLAPS can be updated by re-estimating the relevant statistical components and recalibrating the simulator for contemporary policy evaluation and external validation due to its modular nature. Finally, the simulation model does not distinguish relisted candidates by sensitization or related immunologic factors that can affect donor compatibility and subsequent offer/acceptance dynamics. Future iterations of MoLAPS should explicitly incorporate these developments to accurately reflect evolving transplantation practices and outcomes.

Our findings identify several promising avenues for future research. An immediate next step involves integrating advanced hyperparameter optimization techniques, such as surrogate-based or machine learning methods, to systematically refine policy parameters. Such optimization could improve policy configurations across multiple objectives within the Continuous Distribution framework. Another promising area is evaluating alternative allocation policies, including incentive mechanisms aimed at improving donor-recipient matching performance, reducing organ nonuse, or alleviating existing discrepancies in organ access. The modular structure of MoLAPS facilitates testing such policy designs. Additionally, incorporating detailed logistical factors, including organ transportation times, associated costs, preservation technologies, and regional population densities, could further strengthen the model’s capacity for comprehensive policy analysis. For example, while the current CAS framework considers donor and recipient locations, explicitly accounting for regional demographic and logistical variation would allow more precise evaluations of allocation efficiency and fairness. A further extension is to incorporate economic evaluation, such as cost-effectiveness or cost–benefit analyses, once suitable cost inputs (e.g., procurement/transport) are available and mapped to simulated outcomes. Bayesian calibration is another promising direction for parameter tuning and uncertainty quantification; however, for high-dimensional stochastic DES models it can be computationally demanding because likelihood evaluation typically requires many simulation replications, making it a valuable but technically challenging avenue for future work.

In conclusion, our research delivers a robust, adaptable, and computationally efficient simulation model that significantly advances lung allocation policy analysis. By overcoming critical limitations inherent in previous simulation efforts, MoLAPS provides a valuable decision-support tool for policymakers and researchers, enabling comprehensive exploration of complex policy scenarios and supporting data-driven allocation decisions. Continued refinement of this model, in parallel with ongoing developments in organ allocation practices and technologies, promises meaningful progress toward fairer, more effective lung transplantation systems; ultimately improving patient outcomes and saving more lives.

## Supplementary Information

Below is the link to the electronic supplementary material.

Supplementary file 1 (pdf 534 KB)

## Data Availability

We are not authorized to share the data that we are provided, as our data use agreement (DUA) signed with Organ Procurement and Transplantation Network (OPTN) explicitly states: “You will neither release nor permit others to release the files or data therein to any person (including media and subcontractors) except with the written approval of UNOS.” However, a description of the data can be found at https://www.hrsa.gov/optn/data-calculators/about-optn-data. Data dictionary can be found at https://www.srtr.org/docs/datadictionary.html.
