# Supplementary material for: A comprehensive simulation framework for evaluating U.S. lung allocation policies
Source: Health Care Manag Sci. 2026 Nov 7;29(4):54. doi: 10.1007/s10729-026-09797-z (PMC13744460; doi:10.1007/s10729-026-09797-z)
Supplement: Supplementary file 1 [file 10729_2026_9797_MOESM1_ESM.pdf]

# Appendix A Supplementary Modeling Details

This appendix provides technical details for MoLAPS and a comparison with recent pre-CAS data. Specifically, Section A.1 describes the methodology for predicting future trajectories of patients who received lung transplants in the real-world but remain on the simulated waiting list ("unobserved" cases). Section A.2 explains the discrete-time Markov-chain framework employed for updating LAS, WLAUC, and PTAUC in the absence of empirical data. Section A.3 summarizes the empirical distributions underlying these updates. Section A.4 describes the calibration procedure. Finally, Section A.5 reports a descriptive comparison with empirical outcomes from March 2022 through February 2023.

## A.1 Patient Status Updates for Unobserved Cases

To dynamically model patient survival statuses for individuals transplanted in reality but remaining on the simulated waiting list beyond their actual transplant dates, MoLAPS utilizes a Cox proportional hazards regression model. The survival probability for a patient, conditioned on a vector of covariates $X$, is given by:

$$S(t|X) = e^{-H_0(t)e^{X\beta}},$$

where $S(t|X)$ represents the survival probability at time $t$, $H_0(t)$ denotes the baseline cumulative hazard function, $X$ is the vector of patient-specific covariates detailed in Table S1, and $\beta$ captures the regression coefficients reflecting the influence of each covariate on patient survival. Covariates were selected using a stepwise procedure informed by both clinical expertise and the Akaike Information Criterion (AIC), and included key demographic, clinical, and medical urgency variables known to significantly influence waitlist survival.

**Table S1**: Covariates used in the Cox regression model

| Variable | Type |
|---|---|
| Diagnosis group | Categorical |
| Gender | Binary |
| Initial age | Continuous |
| Height (cm) | Continuous |
| Weight (kg) | Continuous |
| Prior cardiac surgery | Binary |
| O2 requirement at rest | Continuous |
| Hemodynamic cardiac output (L/min) | Continuous |
| FEV1 % predicted | Continuous |
| FVC % predicted | Continuous |
| Central venous pressure (mmHg) | Continuous |
| Six-minute walk distance | Continuous |
| History of cigarette use | Binary |
| Steroid use | Binary |
| LAS | Continuous |

## A.2 Markov Chain Model for Dynamic Score Updates

MoLAPS employs discrete-time Markov chains to dynamically update patient scores (LAS, WLAUC, and PTAUC) when empirical updates are unavailable. The transition probabilities between states $i$ and $j$ are derived from historical data and defined as:

$$P_{ij} = P(X_{t+1} = j | X_t = i), \quad \forall i, j \in \mathcal{S},$$

where $X_t$ denotes the patient state at time $t$, and $\mathcal{S}$ represents the discretized state space. The state spaces for WLAUC and PTAUC range from 0 to 365 days, capturing the full plausible spectrum of patient scores. MoLAPS applies these transition probabilities over seven-day intervals, calculated by iteratively applying the one-day transition matrices. Each transition employs Monte Carlo sampling, generating a uniform random variable on the interval (0,1) to determine subsequent patient states. When historical patient updates are available, the simulation model directly incorporates these recorded transitions to ensure precise alignment with observed patient trajectories.

## A.3 Empirical Waitlist Updates

MoLAPS utilizes detailed empirical data to update patient statuses whenever such records are available to reflect realistic clinical and operational dynamics of the lung allocation process.

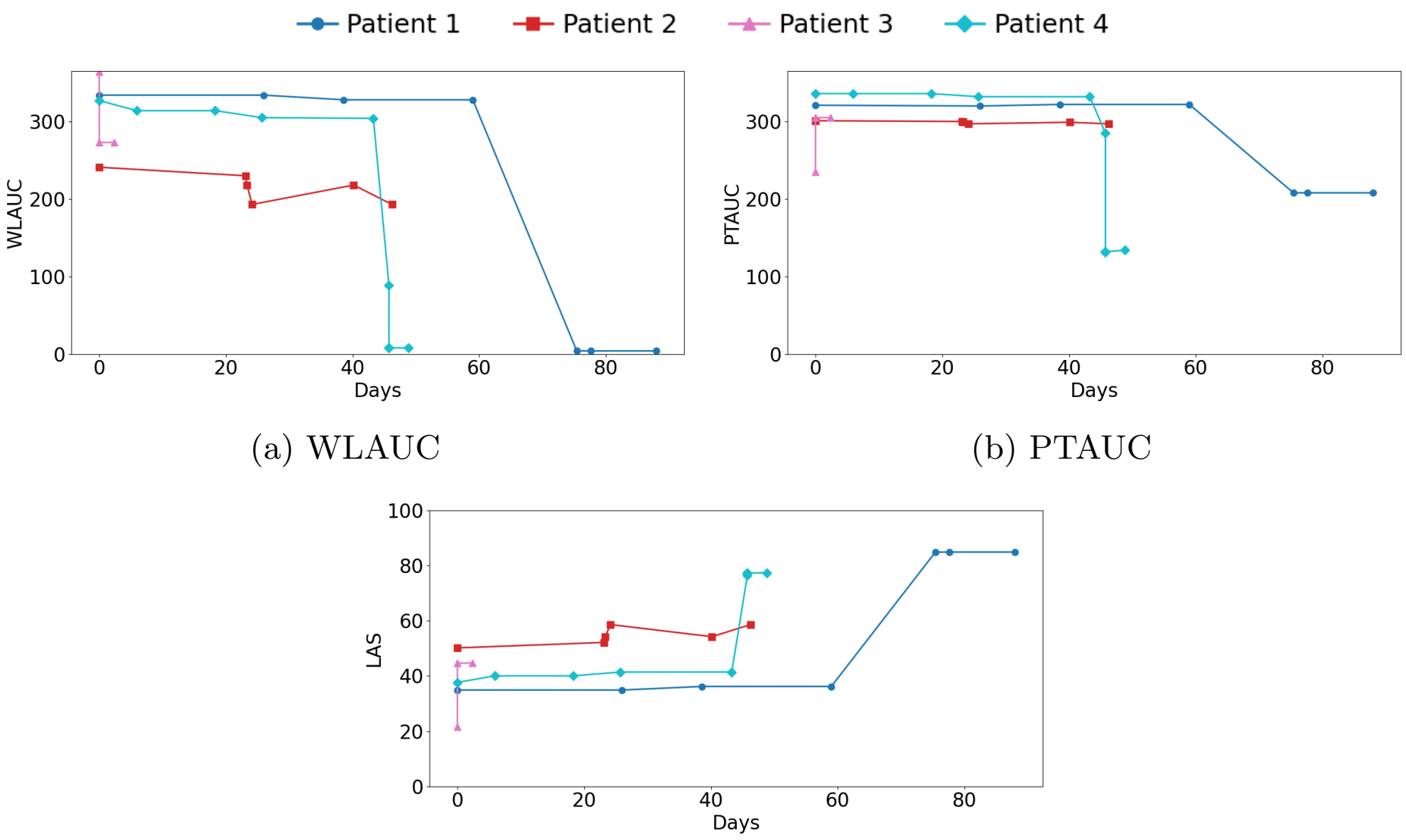


(a) WLAUC

(b) PTAUC

(c) LAS

**Fig. S1**: Evolution of WLAUC, PTAUC, and LAS for selected patients

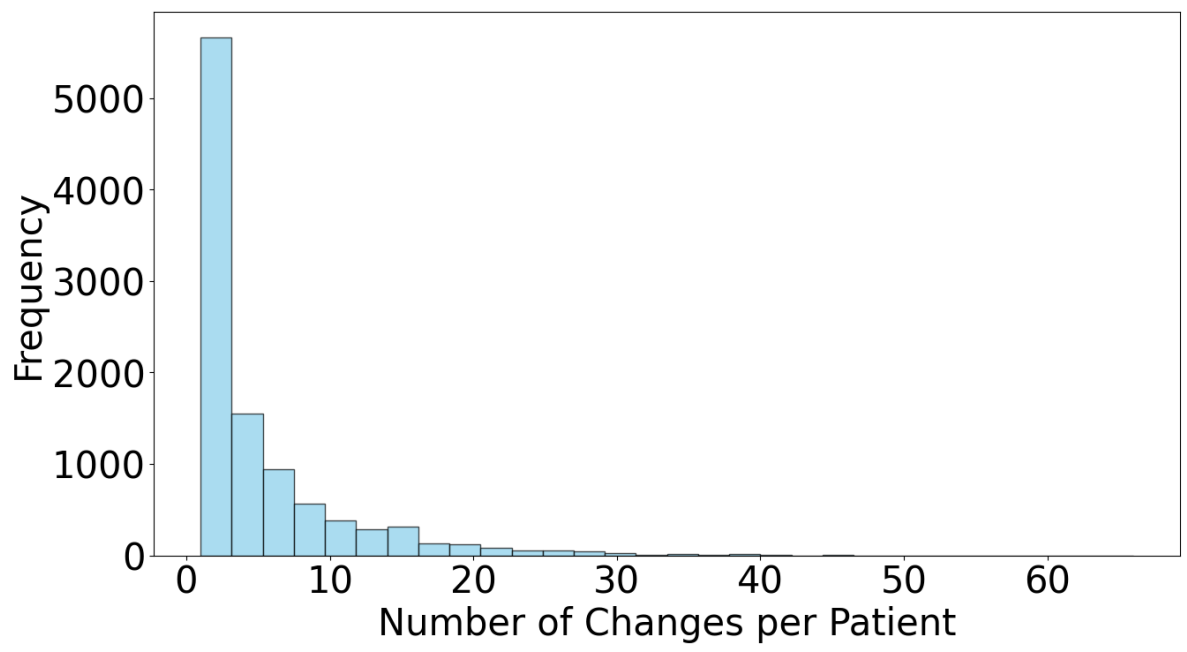


**Fig. S2**: Histogram of the number of empirical waitlist updates per patient

Figure S1 visualizes the evolution of WLAUC, PTAUC, and LAS scores over time for representative patients. These patients were selected using k-means clustering based on the statistical characteristics (moments) of their score trajectories to illustrate diverse patterns of clinical progression.

Additionally, Figure S2 presents the empirical distribution of LAS updates recorded for each patient. The histogram illustrates considerable variability, with most patients receiving fewer than ten updates throughout their waitlist time, although some individuals experience significantly more frequent updates. Instead of imposing a uniform update frequency, MoLAPS directly leverages these empirical data points.

## A.4 Calibration Methodology and Process

The calibration methodology treats the simulation adjustment process as an optimization, searching for parameter values that minimize the deviation between simulation outcomes and observed data. Formally, this optimization involves a function $f : \mathbb{R}^6 \rightarrow \mathbb{R}^4$, mapping the six-dimensional parameter input vector to the four-dimensional output. To quantify the differences between simulated and actual outcomes, we adopt a Euclidean distance metric:

$$d(\Theta, Y_{obs}) = \|\bar{Y}_r(\Theta) - Y_{obs}\|_2,$$

where $\Theta$ denotes the parameter vector, $\bar{Y}_r$ is the sample mean of the simulation outputs with $r$ replications and $Y_{obs}$ the empirical observations. Due to the larger scale of the offer-limit parameter compared to the others, we scale it by dividing by 1,000 prior to calibration to maintain balance in the optimization.

Our calibration employs an iterative surrogate-based optimization process that integrates several complementary approaches. First, we utilize Gaussian Process Regression (GPR) to build a computationally efficient surrogate model capturing input-output relationships across the parameter space [1, 2]. GPR provides probabilistic predictions, enabling extensive exploration without the computational burden of exhaustive simulations. Second, we apply gradient-based optimization using first-order Taylor series approximations to locally refine the parameter search, efficiently guiding

adjustments toward optimal parameter values [2, 3]. Lastly, random search methods introduce controlled stochastic perturbations informed by gradient information to avoid local minima, thereby ensuring robust, global exploration of the parameter space [4].

The calibration process proceeds iteratively, beginning with an initial parameter set and corresponding simulation results. Using the surrogate model and gradient approximations, new candidate parameter sets are proposed and evaluated through additional simulation runs. These new outputs are compared against empirical data, and the best-performing parameters are selected based on the minimum Euclidean distance. This iterative cycle continues until the discrepancy between simulated outputs and empirical data falls below a predefined threshold, $\epsilon > 0$, indicating sufficient calibration accuracy. Algorithm S1 details the iterative calibration procedure explicitly, combining surrogate modeling, gradient approximation, and randomized exploration to systematically identify optimal parameter settings.

After primary calibration, we address a particular category of discrepancies concerning patients who were transplanted in empirical data but remain on the waiting list within the simulation. These deviations represent atypical simulation trajectories requiring targeted adjustments. Specifically, we modify the Cox regression predictions for these patients' waiting times and removal reasons (e.g., death or deterioration), aligning simulated outcomes more closely with observed historical patterns. This adjustment improves the model's fidelity in capturing clinically significant patient pathways, as well as its overall predictive accuracy and reliability.

## A.5 Comparison with a Recent Pre-CAS Period

We compare MoLAPS outputs with empirical outcomes from March 2022 through February 2023, a 12-month period preceding CAS implementation. The simulation uses patient and donor records and waitlist updates from this period while retaining the allocation configuration and calibrated parameters used for 2017–2019. This retrospective comparison examines how the historical configuration performs with later inputs, without recalibration to the later outcomes. The period follows the 2021 revision of the LAS survival models [5], but the analysis does not separately estimate the effects of policy or practice changes. The 2017–2019 simulation results are included only as a historical reference: their three-year horizon differs from the 12-month horizon of the later comparison, so counts and end-of-horizon outcome proportions should not be compared directly across periods.

Table S2 reports outcome counts and proportions. For March 2022–February 2023, the simulated transplant count is 2,752.29 compared with 2,709 observed, and the corresponding transplant proportions are 67.38% and 66.38%. Non-transplant removals are overpredicted (717.77 vs. 471; 17.57% vs. 11.54%), while fewer candidates remain on the waiting list at the end of the horizon (614.57 vs. 901; 15.05% vs. 22.08%). Medically unsuitable removals show the largest difference in composition within non-transplant removals (39.35% vs. 30.36%). Simulated deaths also exceed the observed count (152.93 vs. 116, or 31.84% higher); their smaller share of non-transplant removals (21.31% vs. 24.63%) reflects the larger simulated number of removals overall, not fewer deaths. Organ nonuse is underpredicted (293.71 vs. 337; 9.64% vs. 11.06%).

**Algorithm S1** Iterative Surrogate-based Calibration Procedure

**Require:** Initial dataset of parameter-output pairs $\mathcal{D} = \{(\Theta_i, y_i)\}_{i=1}^{N}$ where $\Theta_i \in \mathcal{H} \subset \mathbb{R}^6$ (calibration parameters), $y_i \in \mathbb{R}^4$ (simulation outputs), $\mathcal{H}$ is the feasible input parameter space.

**Require:** $\Theta^* \in \mathbb{R}^6$: Initial best parameter set (with corresponding output $y^*$).

**Require:** $t \in \mathbb{R}^4$: Target empirical data vector.

**Require:** $\epsilon > 0$: Calibration convergence threshold.

**Require:** $d(\cdot, \cdot)$: Euclidean distance function.

Initialize the hyperparameters for the GPR model $\hat{f}$.

Let $(\Theta_{\text{latest}}, y_{\text{latest}})$ be the last added sample in $\mathcal{D}$.

**while** $d(y^*, t) > \epsilon$ **do**

  Fit surrogate model $\hat{f}$ using GPR with current dataset $\mathcal{D}$.

  Compute an approximate gradient of the distance at the current best point $\Theta^*$:

$$g^* \approx \frac{\Theta^* - \Theta_{\text{latest}}}{d(y^*, t) - d(y_{\text{latest}}, t)}.$$

  Generate new candidate points:

- Candidate 1 (Surrogate minimum):

$$\Theta^{(1)} = \arg\min_{\Theta \in \mathcal{H}} d\left(\hat{f}(\Theta), t\right)$$

- Candidate 2 (Gradient-based step):

$$\Theta^{(2)} = \Theta^* - \beta g^*, \quad \beta > 0 \quad \text{(a small step size)}$$

- Candidate 3 (Randomized exploration):

$$\Theta^{(3)} = \Theta^* - \alpha g^* + z, \quad z \sim N(0, \sigma^2 I)$$

  Run the simulation model at $\{\Theta^{(1)}, \Theta^{(2)}, \Theta^{(3)}\}$ and obtain corresponding outputs $\{y^{(1)}, y^{(2)}, y^{(3)}\}$.

  Update the latest pair based on $y_k$, $k \in \{1, 2, 3\}$

$$(\Theta_{\text{latest}}, y_{\text{latest}}) \leftarrow \arg\min_{k \in \{1,2,3\}} d\left(y^{(k)}, t\right)$$

  Update the simulation dataset: $\mathcal{D} \leftarrow \mathcal{D} \cup \{(\Theta^{(1)}, y^{(1)}), (\Theta^{(2)}, y^{(2)}), (\Theta^{(3)}, y^{(3)})\}$

  Update the current best pair $(\Theta^*, y^*)$ based on the distance between the simulation outputs ($y$) and the original data (t):

$$(\Theta^*, y^*) \leftarrow \arg\min_{(\Theta, y) \in \mathcal{D}} d(y, t)$$

**end while**

**Table S2**: Outcome counts and proportions for the recent pre-CAS period

| | 2017–2019 | March 2022–February 2023 | |
|---|---|---|---|
| Category | Simulation mean | Original data | Simulation mean |
| Macro-level outcomes | | | |
| TX | 7,345.10 (71.32%) | 2,709 (66.38%) | 2,752.29 (67.38%) |
| Non-TX | 1,621.37 (15.74%) | 471 (11.54%) | 717.77 (17.57%) |
| LSWL | 1,332.54 (12.94%) | 901 (22.08%) | 614.57 (15.05%) |
| Micro-level outcomes (within Non-TX) | | | |
| Death | 506.69 (31.25%) | 116 (24.63%) | 152.93 (21.31%) |
| Other removal | 556.05 (34.30%) | 173 (36.73%) | 230.43 (32.10%) |
| Medically unsuitable | 451.95 (27.87%) | 143 (30.36%) | 282.45 (39.35%) |
| Condition improved | 106.68 (6.58%) | 39 (8.28%) | 51.96 (7.24%) |
| Organ nonuse | | | |
| Nonuse | 620.90 (7.79%) | 337 (11.06%) | 293.71 (9.64%) |

Counts are shown with percentages in parentheses. LSWL: last snapshot in the waiting list.

Table S3 reports mean waiting times. Mean time to transplantation is 102.93 days in the simulation and 105.34 days in the empirical data; mean time to death is 149.16 and 147.24 days, respectively. The largest relative discrepancy concerns medically unsuitable removals, for which the simulated mean is 167.71 days compared with 274.64 days observed (38.93% shorter). Mean waiting time across all non-transplant removals is also shorter in the simulation (232.88 vs. 285.90 days).

Together, these results show that close agreement in transplant volume and mean time to transplantation does not extend to all waitlist outcomes. National data document longer-term shifts toward older candidates and restrictive lung disease, greater use of ECMO support at transplantation among adult recipients in 2022 than compared to 2017-2019, and the September 2021 revision of the LAS survival models [6]. These developments provide context for reassessing the historical patient-status and removal models. Although the recent input records capture observed candidate characteristics and waitlist updates, the simulation still relies on historical statistical relationships when empirical follow-up is unavailable. Updating input records alone does not ensure that these relationships remain applicable under later clinical practice.

In particular, the overprediction of medically unsuitable removals and their shorter simulated mean waiting time suggest that the modeling of non-transplant pathways warrants reassessment. Evolving use of bridging support and clinical decisions about continued transplant eligibility could affect how long selected critically ill candidates remain on the waiting list. As discussed in §3.2, recorded removal reasons also depend on center-level assessment and documentation, which may contribute to differences in the composition of non-transplant removals. However, reclassification among removal reasons alone would not explain the excess total removals and smaller end-of-horizon waiting list. The comparison therefore highlights the importance of evaluating both the timing and overall frequency of removals, rather than focusing only on individual removal categories.

On the donor side, national reports document increasing use of donation after circulatory death and higher nonuse of recovered lungs in the years leading to 2022 [6]. Changes in donor assessment and acceptance practices could affect the applicability of historical acceptance parameters and the calibrated offer limit, even when recent donor characteristics are supplied as inputs. The residual underprediction of nonuse makes these components additional priorities for reassessment. Overall, this comparison demonstrates why updating input records and reproducing transplant activity are not sufficient, by themselves, to ensure agreement across all outcomes. It provides a practical basis for targeted updating of patient-removal and offer-acceptance components within MoLAPS's modular architecture before evaluating policies in a later operating environment.

**Table S3**: Mean waiting times for the recent pre-CAS period

| | 2017–2019 | March 2022–February 2023 | |
|---|---|---|---|
| Category | Simulation mean | Original data | Simulation mean |
| TX | 131.00 (−2.20%) | 105.34 | 102.93 (−2.28%) |
| Non-TX | 443.14 (+2.31%) | 285.90 | 232.88 (−18.55%) |
| Death | 225.65 (−6.69%) | 147.24 | 149.16 (+1.30%) |
| Other removal | 617.67 (+8.75%) | 333.86 | 296.06 (−11.32%) |
| Medically unsuitable | 299.95 (+1.98%) | 274.64 | 167.71 (−38.93%) |
| Condition improved | 1,174.97 (−3.23%) | 526.95 | 555.80 (+5.47%) |

Units: days. Parentheses report relative errors against empirical means for the corresponding period.

# References


[1] Gramacy RB. Surrogates: Gaussian Process Modeling, Design and Optimization for the Applied Sciences. Boca Raton, Florida: Chapman Hall/CRC; 2020. http://bobby.gramacy.com/surrogates/.

[2] Xu J. Model calibration. In: Advances in Modeling and Simulation: Seminal Research from 50 Years of Winter Simulation Conferences. Springer; 2017. p. 27–46.

[3] Yuan J, Ng SH, Tsui KL. Calibration of stochastic computer models using stochastic approximation methods. IEEE Transactions on Automation Science and Engineering. 2013;10(1):171–186. https://doi.org/10.1109/TASE.2012.2199486.

[4] Bergstra J, Bengio Y. Random search for hyper-parameter optimization. The Journal of Machine Learning Research. 2012;13(1):281–305.

[5] Valapour M, Lehr CJ, Schladt DP, Smith JM, Goff R, Mupfudze TG, et al. OPTN/SRTR 2021 Annual Data Report: Lung. American Journal of Transplantation. 2023 feb;23(2 Suppl 1):S379–S442. https://doi.org/10.1016/j.ajt.2023.02.009.

[6] Valapour M, Lehr CJ, Schladt DP, Smith JM, Swanner K, Weibel CJ, et al. OPTN/SRTR 2022 annual data report: lung. American Journal of Transplantation. 2024;24(2):S394–S456.
